# Diet Is a Stronger Covariate than Exercise in Determining Gut Microbial Richness and Diversity

**DOI:** 10.3390/nu14122507

**Published:** 2022-06-16

**Authors:** Eon-Joo Yun, Saba Imdad, Junho Jang, Jinhan Park, Byunghun So, Jin-Hee Kim, Chounghun Kang

**Affiliations:** 1 Molecular Metabolism in Health & Disease, Exercise Physiology Laboratory, Sport Science Research Institute, Inha University, Incheon 22212, Korea; eonjooyun@gmail.com (E.-J.Y.); jangju2489@gmail.com (J.J.); sportsjinhan@gmail.com (J.P.); sportshun@gmail.com (B.S.); 2 Department of Biomedical Laboratory Science, College of Health Science, Cheongju University, Cheongju 28503, Korea; jinheekim@cju.ac.kr; 3 Department of Physical Education, College of Education, Inha University, Incheon 22212, Korea

**Keywords:** treadmill exercise, high-fat diet, mouse gut microbiome, QIIME 2, 16S rRNA amplicon sequencing, next-generation sequencing (NGS), microbiota diversity and richness

## Abstract

Obesity is a common metabolic disorder caused by a sedentary lifestyle, and a high-fat and a high-glucose diet in the form of fast foods. High-fat diet-induced obesity is a major cause of diabetes and cardiovascular diseases, whereas exercise and physical activity can ameliorate these disorders. Moreover, exercise and the gut microbiota are known to be interconnected, since exercise can increase the gut microbial diversity and contribute to the beneficial health effects. In this context, we analyzed the effect of diet and exercise on the gut microbiota of mice, by next-generation sequencing of the bacterial V4 region of 16S rRNA. Briefly, mice were divided into four groups: chow-diet (CD), high-fat diet (HFD), high-fat diet + exercise (HFX), and exercise-only (EX). The mice underwent treadmill exercise and diet intervention for 8 weeks, followed by the collection of their feces and DNA extraction for sequencing. The data were analyzed using the QIIME 2 bioinformatics platform and R software to assess their gut microbial composition, richness, and diversity. The Bacteroidetes to Firmicutes ratio was found to be decreased manifold in the HFD and HFX groups compared to the CD and EX groups. The gut microbial richness was comparatively lower in the HFD and HFX groups and higher in the CD and EX groups (ACE, Chao1, and observed OTUs). However, the Shannon alpha diversity index was higher in the HFD and HFX groups than in the CD and EX groups. The beta diversity based on Jaccard, Bray–Curtis, and weighted UniFrac distance metrics was significant among the groups, as measured by PERMANOVA. *Paraprevotella*, *Desulfovibrio*, and *Lactococcus* were the differentially abundant/present genera based on the intervention groups and in addition to these three bacteria, *Butyricimonas* and *Desulfovibrio C21c20* were differentially abundant/present based on diet. Hence, diet significantly contributed to the majority of the changes in the gut microbiota.

## 1. Introduction

The obesity pandemic has been the largest contributor to ill health according to the World Health Organization (WHO) [1]. According to the latest WHO fact sheets, more than 1.9 billion adults and 600 million individuals have been classified as overweight or obese worldwide, respectively, and these rates are projected to increase at a faster pace in the coming decades [2]. Obesity is caused by a combination of genetic and environmental factors including genetic predisposition, the disruption of energy metabolism, and environmental and social factors [3]. A high-fat diet (HFD) and inadequate physical activity are major environmental factors causing obesity, while genetics also plays an important role in terms of body weight homeostasis [4]. Obesity is, therefore, expected to place a serious burden on the public healthcare system. Coronary artery disease risk factors and obesity are positively correlated [5]. Obesity causes several changes in the body, including the induction of inflammation, oxidative stress, mitochondrial dysfunction, and apoptosis [6]. Specifically, morbid obesity is associated with hypertension, type 2 diabetes, and venous thromboembolism [7].

Microorganisms are present on and in the human body, thriving on the skin and in the genitourinary, gastrointestinal, and respiratory tracts, and constitutes our microbiota, which is dominated by 2000 anaerobic bacteria [8,9]. Host nutrition, metabolic function, and maturation of the immune system are greatly benefited by healthy microbiota [10,11]. A more comprehensive analysis of the gut microbiota has been enabled by the development of next-generation sequencing (NGS) technologies. Recently, a National Institute of Health (NIH), USA, project revealed the presence of more than 70 bacterial phyla in the gut microbiota, with the majority belonging to *Actinobacteria*, *Bacteroidetes*, *Firmicutes*, and *Proteobacteria* [12]. Another study revealed that obesity was associated with changes in the composition and metabolic function of the gut microbiota in diet-induced obese and knockout mice [13]. Studies have shown that there are diverse numbers of microbial species in the distal gastrointestinal tract, and the balance between the composition and function of these intestinal microbes is disturbed during gut dysbiosis [14].

The host physiology is heavily dependent on the gut microbiota, and the host metabolism may be affected by differences in gut microbial composition [15]. Population [16,17,18,19] and clinical studies [20] have identified various factors that cause changes in the human microbiome, illustrating that microbiota are closely associated with disease and health. For instance, the childbirth delivery method [21], use of antibiotics, physical activity, and diet can regulate the gut microbial composition, where diet can explain over 50% of the changes in the gut microbiota [22]. Diet has been indicated as a key determinant of gut microbiome composition, greatly influencing the selection of the gut microbiota [15].

Moreover, endurance exercise can also benefit human health by the diversification of the gut microbiota [23,24]. Numerous chronic diseases, including obesity, can be prevented or ameliorated by regular physical exercise [25]. Exercise may help reduce inflammation, improve body composition, and nurture the gut microbial diversity that contributes to an efficient metabolism [26]. Several controlled animal experiments have demonstrated that the composition and functional capacity of the gut microbiota can be altered by physical exercise alone [27]. VO_2_ max, the maximum rate of oxygen consumption, is a measure of physical fitness and it was found to be correlated with the Bacteroidetes/Firmicutes (B/F) ratio [28]. Although most studies have shown an increase in the B/F ratio following physical activity and exercise [29,30,31], some studies have instead reported a decrease in this ratio [26,32,33]. Recent studies have shown that aerobic exercise results in a larger diversity and abundance of genera from the *Firmicutes* phylum, which could explain why exercise positively affects the gut and the brain [34]. Elite rugby players showed a higher diversity of gut microbiota compared to other individuals, and the differences in the microbiota of these players were shown to be associated with decreased inflammation [26]. Health-promoting bacteria were found to be increased in women performing the minimum amount of exercise regularly, as recommended by the WHO, and it had a positive effect on their body fat percentage and muscle mass [35]. In a study evaluating the physical endurance of mice using swimming as an index of exercise capacity, the germ-free mouse model showed lower exercise capacity and decreased endurance when compared to the mice harboring a single bacterial species in their guts [36].

There have been many studies on the factors that affect the microbiome, such as physical exercise and HFD. However, the effect of exercise in combination with diet intervention, such as HFD, on the composition of the gut microbiome, is not well understood. In this study, we aimed to understand the covariates responsible for the changes in the gut microbiota, using diet (involving chow and HFD) and treadmill exercise interventions, in the context of diet-induced obesity.

## 2. Materials and Methods

### 2.1. Animal and Fecal Sample Collection

**Figure 1:**
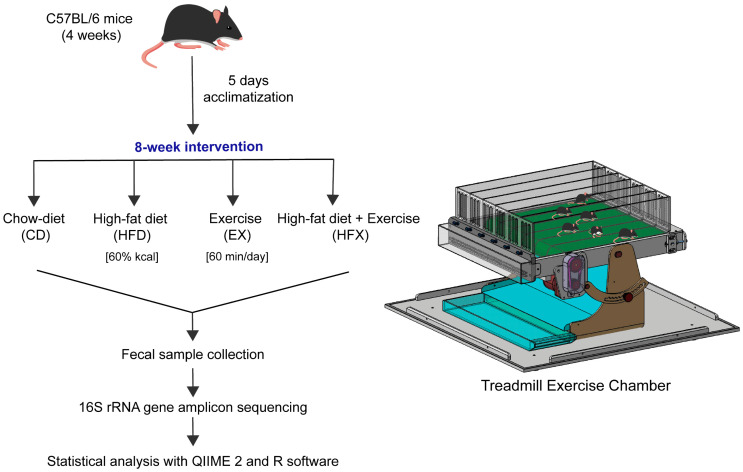
Research design for the analysis of the mouse gut microbiome using 16S rRNA gene amplicon sequencing.

Four-week-old C57BL/6 female mice (n = 5) were randomly assigned to four groups, namely chow diet (CD), high-fat diet (HFD), high-fat diet + exercise (HFX), and exercise-only (EX) groups. Normal mice were fed a standard rodent chow diet (protein 18%, fat 5%, fiber 5%, ash 5% of total weight) and HFD mice were fed a high-fat diet (protein 20.5%, fat 34.9%, fiber, ash 8.0%, calcium, phosphorus 0.5% of total weight). The mice were housed in sterile cages, at a temperature of 22 ± 2 °C and relative humidity of 50 ± 10%, under 12 h light/dark cycle. After 8 weeks of diet and treadmill exercise intervention, the mice were fasted for 12 h and sacrificed by isoflurane overdose in a random order. The body weight of the mice was recorded every week until the last week. Fecal samples from individual mice were collected by restraint, in sterile microcentrifuge tubes, snap frozen, and stored at −80 °C, until further processing. The design of the study is presented in Figure 1.

### 2.2. Exercise Protocol

The mice were housed in a cage for a week and adapted to treadmill exercise for five days prior to the start of the exercise experiment. Exercise was performed for 8 weeks in a treadmill chamber. Mice from the EX and HFX groups underwent treadmill exercise for a duration of 60 min/day on a 5% slope, with a gradually increasing intensity from 4 m/min to 15 m/min for the initial 30 min, then increasing to 20 m/min for another 30 min, followed by 5 min of cooling at 5 m/min.

### 2.3. DNA Extraction

The mouse fecal samples (frozen) were thawed at 4 °C and weighed (200 mg) for DNA extraction by SPINeasy DNA Kit for Feces (MP Biomedicals, Irvine, CA, USA), according to the manufacturer’s instructions. Briefly, the stool samples were homogenized using a Fast-Prep 24 (MP Biomedicals, Irvine, CA, USA) bead homogenizer in tubes prefilled with high-quality beads, which ensured the disruption of the cell walls and the release of DNA into the solution. The extracted DNA was examined for integrity using electrophoresis on 1% agarose gel and visualized using ChemiDoc (Bio-Rad, Hercules, CA, USA).

### 2.4. DNA Quantification

DNA quantification and quality measurements were performed using two spectrophotometric devices, Spectra Max iD3 (Molecular Devices, San Jose, CA, USA) and Qubit 4 (Thermo Fisher Scientific, Waltham, MA, USA), based on the absorbance and fluorescence readouts, respectively. The A260/A280 absorbance ratio determined using Spectra Max iD3 was used to estimate the quality and purity of the extracted DNA, while the concentration was measured using Qubit 4.

### 2.5. 16S rRNA-Based Amplicon Sequencing 

The extracted DNA was amplified using barcoded primers that targeted the bacterial V4 region of the 16S rRNA gene. We used V4 region-specific primers along with locus-specific overhang sequences for PCR. The sequences used were 515F-5′-TCGTCGGCAGCGTCAGATGTGTATAAGAGACAG-GTGCCAGCMGCCGCGGTAA- 3′ and 806R -5′-GTCTCGTGGGCTCGGAGATGTGTATAAGAGACAGGGACTACHVGGGTWTCT- AAT-3′. The PCR thermal cycling conditions were 95 °C for 3 min, 72 °C for 5 min, 25 cycles of 95 °C for 30 s, 55 °C for 30 s, and 72 °C for 30 s. We attached the indices and Illumina sequencing adapters through index-PCR using the Nextera XT index kit v2 (Illumina, USA). The PCR thermal cycling conditions for index-PCR were 95 °C for 3 min, 72 °C for 5 min, eight cycles of 95 °C for 30 s, 55 °C for 30 s, and 72 °C for 30 s. PCR clean-up was performed to purify the 16S V4 amplicons using AMPure XP beads (Beckman Coulter, Brea, CA, USA), after every PCR amplification. The samples were normalized, pooled, mixed with the PhiX control v3 (Illumina), and sequenced using the Illumina iSeq 100 platform.

### 2.6. 16S rRNA-Sequencing Analysis

Paired-end FASTQ sequences were imported into QIIME2 v2021.04 [37] and inspected with FastQC for quality control parameters, such as PhiX contamination, read trimming, and adapter sequences. The analysis was carried out using QIIME 2, unless otherwise stated. The PCR primers were removed using Cutadapt v3.4 [38] and the sequences were merged using VSEARCH v2.7.0 [39], followed by quality filtration (Q20) and dereplication. De-novo clustering was performed to select OTUs, followed by chimera removal, and representative sequences and abundances were extracted. A naïve Bayes classifier [40] was trained using 16S rRNA V4 region sequences extracted from the Greengenes 13.8 database [41], and the representative sequences were classified by taxon using the trained classifier. Alpha diversity indices, such as observed OTUs, Chao1, ACE, and Inverse Simpson, were estimated using the phyloseq package v1.38 of R, by importing the data from QIIME 2 in biom format. However, Shannon and Pileou’s evenness were estimated using QIIME 2. Alpha diversity significance was calculated using the Kruskal–Wallis test, followed by the improved Benjamini–Hochberg procedure for false discovery rate (FDR) correction. Principal coordinate analysis (PCoA) was performed based on Jaccard, Bray–Curtis, and weighted UniFrac distance metrics to stratify the samples and determine the microbial community structure. Permutational multivariate analysis of variance (PERMANOVA) was employed to determine the statistical significance of the PCoA results, which were later confirmed by PERMDISP. Longitudinal ANOVA and ADONIS were used to determine the significant covariates of diversity. Analysis of the composition of microbiomes (ANCOM) was employed to determine differentially abundant taxa. The random forest model [42] was used to generate a heatmap of important taxa. Statistical significance was calculated using two-way ANOVA, one-way ANOVA, and Tukey’s post hoc test. The results are expressed as the mean ± SEM. The significance level was set at *p* < 0.05.

## 3. Results

The body weight and food intake of each mouse were recorded once a week for 8 weeks. Table 1 shows the average initial and final body weights of the mice at the end of the experiment. After the 8-week intervention, the HFD group showed a significant increase in the final body weight compared to the CD group (*p* < 0.0001). As expected, the HFD group mice showed a higher body weight than the mice in the EX group (*p* = 0.0003). Similarly, the HFX group mice gained significantly more weight than the CD group mice (*p* = 0.0046). However, the CD and EX groups showed similar weight gain after 8 weeks. HFD can induce weight gain and increase body fat content in mice, as reported previously [43]. The mice in the HFX group weighed less on average compared to HFD-fed mice; however, no significant difference was recorded in weight gain between the HFD and HFX groups. The average food intake of the groups is shown in Table 1, with no significant differences observed among the groups.

### 3.1. Gut Microbial Richness and Diversity

**Figure 2:**
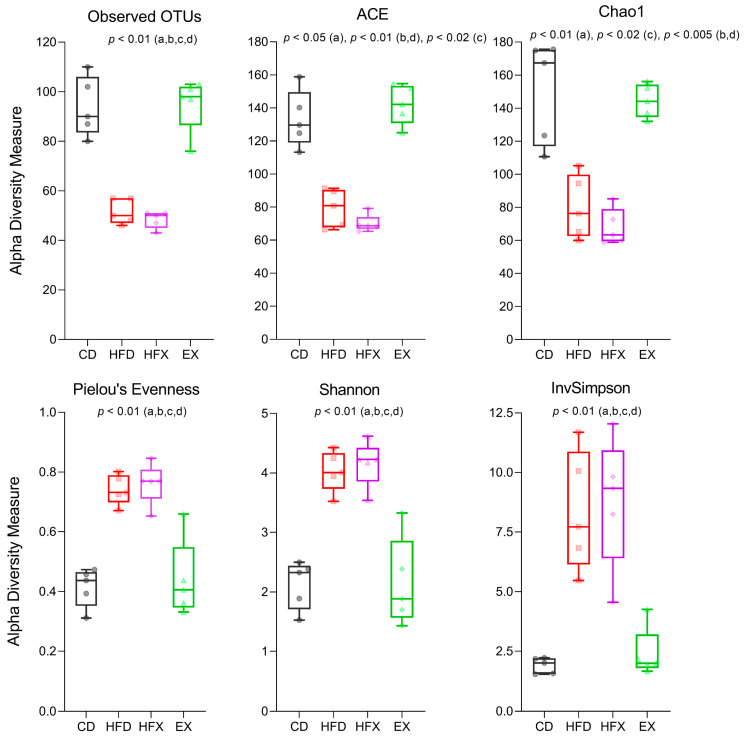
Alpha diversity indices for species richness (observed OTUs, ACE, Chao1), evenness (Pielou’s evenness), and diversity (Shannon, inverse Simpson). Kruskal–Wallis test and two-stage linear step-up procedure of Benjamini, Krieger, and Yekutieli tests for multiple comparisons were performed for median comparison across groups. Boxes denote the interquartile ranges, lines indicate medians, and whiskers demarcate the ranges. *p*-values are FDR corrected. a: CD/HFD, b: CD/HFX, c: HFD/EX, d: HFX/EX.

The gut microbial richness was determined using different alpha diversity indices. The HFX group had low richness as measured by ACE, Chao1 and observed OTUs, where no significant difference was observed compared to the HFD group. In contrast, the CD group showed the highest richness, with no significant difference compared to the EX group (Figure 2). Diversity estimation with the inverse Simpson metric showed patterns and results like those of the richness estimation analysis, with significantly lower diversity in the HFD and HFX groups than in the CD and EX groups (Figure 2). The Shannon diversity did not differ between the HFD and HFX groups, and between the CD and EX groups. Moreover, the Shannon index, which considers richness and evenness, was higher in the HFD and HFX groups than in the CD and EX groups. A similar pattern was observed for Pileou’s evenness (Figure 2).

**Figure 3:**
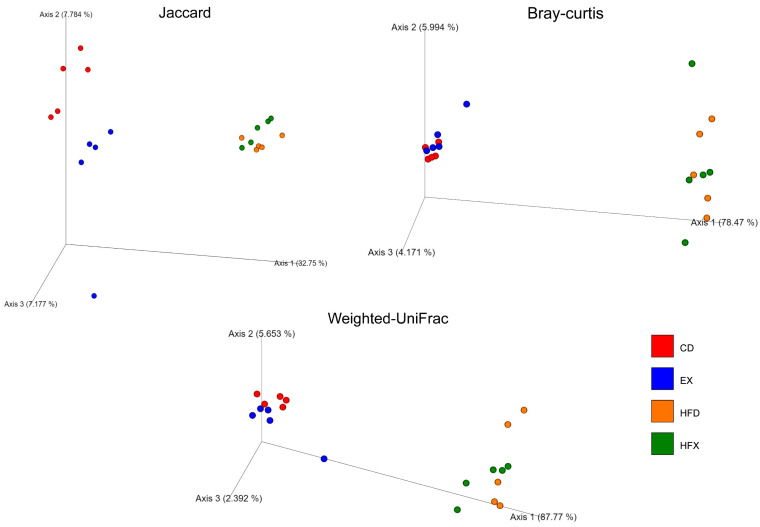
Principal co-ordinate analysis (PCoA) based on Jaccard, Bray–Curtis, and weighted UniFrac metrics for microbial community comparison, at the OTU level. The data points represent individual samples. Statistical significance was determined using PERMANOVA by comparing the true F statistics to the randomly permuted F statistics defaulted to 999 permutations. PERMDISP was used to confirm the significance analysis; Jaccard (*p* = 0.03), Bray–Curtis (*p* = 0.001), and weighted UniFrac (*p* = 0.007).

The structure of the gut microbial community of the different groups was examined using PCoA, and statistical significance was determined using PERMANOVA and confirmed using PERMDISP (Figure 3). There was a partial separation between the HFD and HFX groups along axis 1 and axis 2, in Bray–Curtis and weighted UniFrac distance-based PCoA. Jaccard, which is a qualitative non-phylogenetic beta diversity metric, showed significant differences among all groups (*p ≤* 0.02), except for the HFD and HFX groups (Figure 3). Similarly, the Bray–Curtis metric was found to be significant among the groups (*p* ≤ 0.02, CD/EX, *p* = 0.024), except for the HFD and HFX groups. In contrast, the weighted UniFrac distance metric-based PCoA revealed significant differences among the groups (*p* < 0.02), except for CD/EX and HFD/HFX. Overall, the HFX and HFD groups showed higher variability, while the CD group had the lowest variability in Jaccard and Bray–Curtis based PCoA (Figure 3). Overall, the dissimilarities between the CD and EX groups were evident without overlap in Jaccard and Bray–Curtis-based PCoAs, and with some overlap in the weighted UniFrac-based PCoA.

### 3.2. Microbial Taxonomic Profiling of Mouse Gut

**Figure 4:**
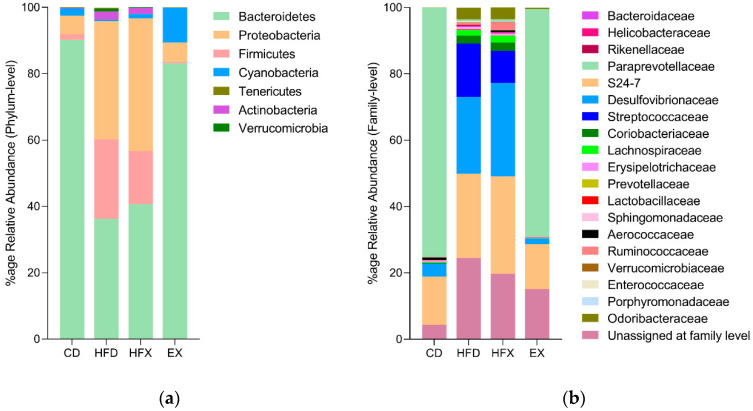
Taxonomic composition of mice gut microbiota at (**a**) phylum level and (**b**) family level. Two-way ANOVA and multiple comparison tests were used to determine significance (*p* < 0.05).

Bacteroidetes and Firmicutes are the two largest phyla in the gut, which together account for approximately 90% of the total gut microbiota. Bacteroidetes accounted for approximately 89% and about 83% of the OTUs in the CD and EX groups (*p* = 0.14, ns), respectively (Figure 4a). Likewise, the profile of Bacteroidetes in the HFD and HFX groups were similar but decreased to approximately half (*p* < 0.0001) of those estimated for the CD and EX groups. Moreover, Firmicutes accounted for about 24% and 16% of the OTUs in the HFD and HFX groups, respectively, with no significant difference between the two groups (Figure 4a). In addition to the increased proportion of Firmicutes in the HFD and HFX groups, Proteobacteria also showed substantial expansion close to 40% in these groups (*p* < 0.0001) compared to the CD and EX groups (Figure 4a). Conversely, the relative abundance of Proteobacteria in the CD and EX groups was below 10%, and the profile was similar between the two groups. The relative abundance of the phylum Cyanobacteria significantly increased by approximately 4.7-fold, 7.8-fold, and ~30-fold in the EX group only, compared to the CD group (*p* = 0.04), HFX group (*p* = 0.02), and HFD group (*p* = 0.009), respectively. Nonetheless, the relative abundance of Verrucomicrobia was rare in the intervention groups (Figure 4a).

*Paraprevotellaceae* was the most abundant bacterial family of the phylum Bacteroidetes in the CD group and was significantly prevalent compared to its negligible proportions in the HFD and HFX groups (*p* < 0.0001) (Figure 4b). The EX group showed a significant decrease in the *Paraprevotellaceae* family compared to the CD group (*p* = 0.01). Another family of Bacteroidetes, *S24-7*, was significantly higher in relative abundance in the high-fat diet-fed groups (HFD and HFX) in comparison to the chow-fed groups (CD and EX) (*p* < 0.0001), regardless of the effect of exercise activity (Figure 4b). Likewise, *Desulfovibrionaceae* of the phylum Proteobacteria showed a similar pattern of relative abundance, with an increased proportion of about 20–30% in the HFD and HFX groups compared to about 2–4% in the CD and EX groups (*p* < 0.0001). *Streptococcaceae*, which belongs to the phylum Firmicutes, was rarely identified in the CD group, but was significantly abundant in the HFD group (~16%) (*p* < 0.0001). Interestingly, the abundance of the *Streptococcaceae* family was significantly reduced in the HFX group ~1.6-fold compared to the HFD group (*p* = 0.01) (Figure 4b). The bacterial taxonomic families responsible for the increased abundance of the phyla Proteobacteria and Firmicutes in the high-fat diet-fed groups (HFD, HFX) were *Desulfovibrionaceae* and *Streptococcaceae*, respectively (Figure 4). In contrast, the reduction of *Paraprevotellaceae* in the high-fat diet-fed groups (Figure 4b) might be the major contributing taxonomic family for the decreased proportion of phylum Bacteroidetes observed in these groups (Figure 4a).

#### Genus/Species Level Classification

**Figure 5:**
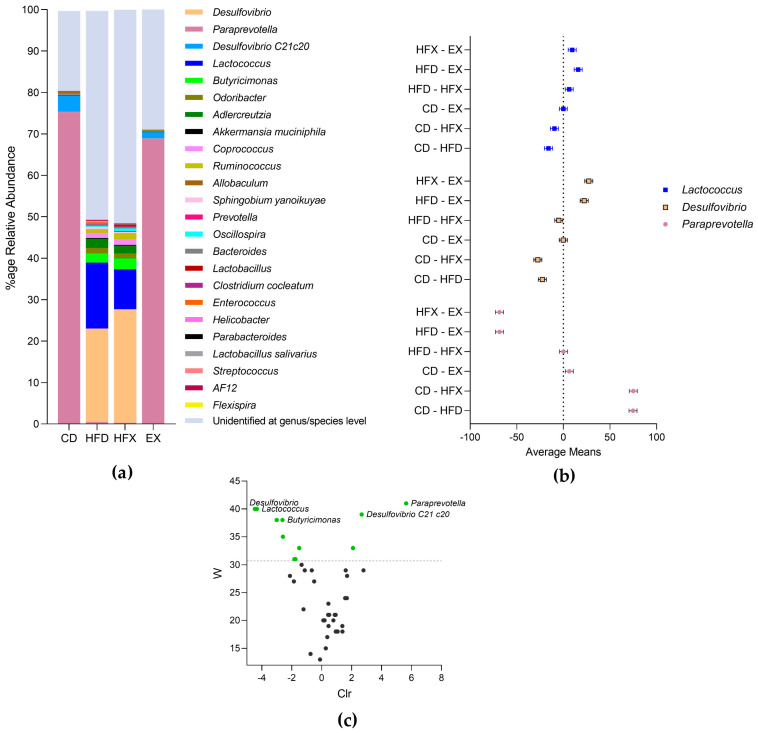
Taxonomic profiling and differentially abundant taxa of the mouse gut microbiota. (**a**) Genus/species level taxonomy. (**b**) Tukey’s multiple comparison analysis of genera (two-way ANOVA) with 95% confidence interval. (**c**) Volcano plot illustrating differentially abundant genera based on diet intervention. The *x*-axis represents clr (centered log ratio)-transformed mean difference in abundance, of the genus level OTUs, while W represents the frequency of null-hypothesis rejection for a given species. The dotted grey line represents the significance threshold (*p* = 0.05).

Among the 30 taxa identified at the genus or species level in the mouse gut (Figure 5a), *Paraprevotella*, *Desulfovibrio,* and *Lactococcus* were significantly altered in the experimental groups, as illustrated in Figure 5b. *Paraprevotella* of the phylum Bacteroidetes was significantly decreased in relative abundance in the EX group (*p* = 0.0006) compared to the CD group. However, its abundance was scarce in the HFD and HFX groups, with no difference between the groups. The decreased percentage of Bacteroidetes in the HFD and HFX groups compared to that in the CD and EX groups can be attributed to the decreased abundance of *Paraprevotella* (Figure 4a and Figure 5a,b). The genus *Desulfovibrio* from the Proteobacteria phylum increased significantly in the HFD and HFX groups compared to its proportion in the CD and EX groups (*p* < 0.0001). Interestingly, the proportion of the *Desulfovibrio* genus was significantly higher in the HFX group than in the HFD group (*p* = 0.017). The relative increase in the proportion of Firmicutes in the HFD and HFX groups (Figure 4a) was reflected at the genus level and is attributed to *Lactococcus. Lactococcus* was significantly reduced in the HFX group ~1.6-fold compared to its proportion in the HFD group (*p* < 0.0009), as shown in Figure 5a, b. The presence of *Lactococcus* was not identified in the CD and EX groups. Moreover, *Bacteroides ovatus, Sutterella, Atherosperma moschatum, Anaeroplasma, Lactobacillus reuteri*, and *Coprobacillus* were identified with negligible relative abundance in all the experimental groups (data not shown). Unidentified species fell in the range of ~20–50% (Figure 5a).

### 3.3. Differentially Abundant and Important Taxa of Mouse Gut Microbiota

ANCOM based on diet revealed the differentially abundant or present taxa in the high-fat diet-fed groups and chow-fed groups. Among these, *Paraprevotella* (*Paraprevotellaceae*, Bacteroidetes) and *Desulfovibrio C21c20* (*Desulfovibrionaceae*, Proteobacteria) were differentially abundant/present in the chow-fed groups (CD, EX), whereas *Lactococcus* (*Streptococcaceae*, Firmicutes), *Desulfovibrio* (*Desulfovibrionaceae*, Proteobacteria) and *Butyricimonas* (*Odoribacteraceae*, Bacteroidetes) were differentially abundant/present in the high-fat diet-fed groups (HFD, HFX) (Figure 5c). Almost half of the microbiota that were significantly altered with respect to the diet intervention belonged to *S24-7* bacterial taxonomic family of the order Bacteroidales, within the phylum Bacteroidetes. Moreover, 80% of *S24-7* family members identified by ANCOM based on diet were relatively significantly abundant in the high-fat diet-fed groups (Figure 5c).

**Figure 6:**
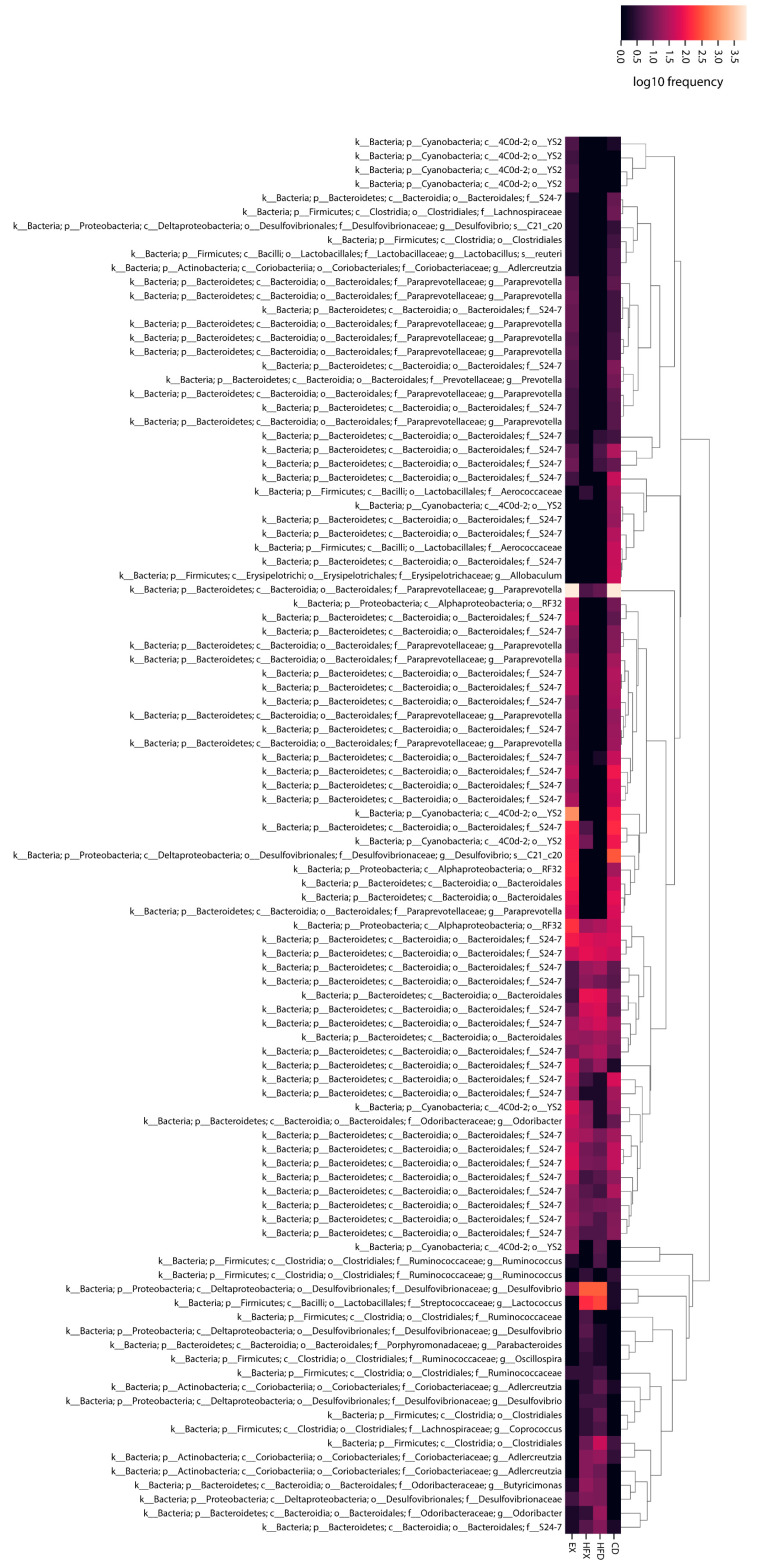
Heatmap showing the most important taxa among experimental groups.

The most important taxa within the OTU table were generated using the random forest machine-learning algorithm (Figure 6). The majority of the members of the order YS2 from the phylum Cyanobacteria were relatively abundant in the EX group compared to the other groups. On the other hand, *Allobaculum* (Firmicutes) was relatively abundant in the CD group, as shown in the heatmap (Figure 6). Among the four *Adlercreutzia* (Actinobacteria) species identified as the important taxa between the intervention groups, three were relatively abundant in the high-fat diet-fed groups (HFD, HFX) (Figure 6). *Oscillospira* (Firmicutes) was insignificantly enhanced in the HFX group compared to the other groups. Two species of *Odoribacter* (Bacteroidetes) were identified with contrasting abundance profiles, where one taxon showed moderately higher abundance in HFD than the other groups, while the other taxon showed strikingly higher abundance in all groups except HFD (Figure 6). *Coprococcus* (Firmicutes) was found to be relatively mildly higher in abundance in the high-fat diet-fed groups. *Desulfovibrio* and *Lactococcus* were abundant in the HFD and HFX groups, while *Prevotella* was relatively frequently observed in the CD and EX groups (Figure 6).

## 4. Discussion

The B/F ratio is known to have an important influence on the homeostasis of the gut microbiome. An altered B/F ratio is associated with dysbiosis and several other pathological conditions [44]; for example, an increase in the proportion of Firmicutes has been observed in obesity, while an increase in the proportion of Bacteroidetes has been observed in inflammatory bowel disease (IBD) [45]. The B/F ratio varies depending on the food source, where a higher relative proportion of Firmicutes is associated with HFD. This can be explained by the efficient extraction of energy from food by the Firmicutes than Bacteroidetes, thereby promoting more efficient absorption of calories by the host and the consequent weight gain [46]. Our results revealed a similar B/F ratio in the CD and EX groups; moreover, no significant difference was observed in the B/F ratio between the HFD and HFX groups. However, the B/F ratio drastically decreased by about 30-fold in the HFD group in comparison to the CD group (Figure 4a). Our results corroborate with the findings of the previous studies, which showed an increased abundance of Firmicutes in obese and HFD-fed mice compared to that in the lean mice [47,48,49]. In agreement with our results, another study showed an increase in the populations of Firmicutes and Proteobacteria in response to the consumption of unsaturated fat (74%) [50]. In the present study, a higher abundance of Proteobacteria was observed in the HFD group (*p* < 0.0001) compared to the CD group; this increased abundance has been reported to be a potential diagnostic marker for dysbiosis and other metabolic diseases [51]. In addition, we observed a significantly increased proportion of Cyanobacteria in the EX group only, compared to all other groups (Figure 4a). Cyanobacteria are primarily recognized as photosynthetic and have presumably shaped the earth’s geochemical environment over some billions of years by generating oxygen as a by-product [52]. The bacterial taxa YS2, which was previously recognized as a member of Cyanobacteria, is now part of another taxa called ‘Melainabacteria’, based on recent genomic evidence that showed the absence of photosynthetic abilities in YS2. The Cyanobacteria-related YS2 demonstrates many functions, such as obligate anaerobic fermentation, nitrogen fixation, and the synthesis of vitamin B and K, as illustrated by the metabolic analysis. YS2 are common in the mammalian gut [53], and in the current study, it was found in higher proportions in the gut of mice that underwent exercise (EX) (Figure 6).

*Paraprevotallaceae* was significantly more abundant in the EX group compared to the CD group. An earlier report showed that this taxon has decreased relative abundance in female obese swine (Western diet-fed) than in lean swine (low-fat diet-fed) after 36 weeks of diet intervention [54]. The result of the present study and the earlier one can be explained by the difference in the diet intervention periods and the choice of experimental animals. The specific majority of the members of the *S24-7* microbiota family that were identified by ANCOM and based on diet showed differential abundance in the high-fat diet-fed groups (HFD, HFX) compared to the chow-fed groups (CD, EX) (Figure 5c). These species might be contributing to the significant expansion of the *S24-7* family in the high-fat-fed groups, as illustrated in Figure 4b. The members of *Desulfovibrionaceae* and *Streptococcaceae* were significantly enhanced in the HFD group, and exercise effectively reversed the proportions of *Streptococcaceae*, as shown by the HFX group in Figure 4b. *Desulfovibrionaceae* include sulphate-reducing and endotoxin-producing bacteria, which are enhanced in HFD [22]. A recent report illustrated the increase of *Desulfovibrionaceae* and *Peptostreptococcaceae* in the HFD-fed mice, which was reversed by exercise [55]. Moreover, *Streptococcaceae* was associated with high-BMI individuals and a high-fat diet intake [56]. These reports support our data in demonstrating the beneficial impact of exercise on the gut microbiota of HFD mice. 

Although *Ruminococcaceae* (Firmicutes), *Lachnospiraceae* (Firmicutes), *Odoribacteraceae* (Bacteroidetes), and *Coriobacteriaceae* (Actinobacteria) did not reach statistical significance, these bacterial taxonomic families were found to be bloomed in the high-fat diet-fed groups compared to the chow-fed groups (Figure 4b). An earlier report showed that the long-term intake of a high-fat diet increases *Lachnospiraceae* and *Streptococcaceae* bacteria in C57BL/6 mice, and inclines towards an increased inflammatory profile [57]. Similarly, at the genera level, *Coprococcus* (*Lachnospiraceae*) and *Aldercreutzia* spp. (*Coriobacteriaceae*) did not attain statistical significance but were found to be intensified in the high-fat diet-fed groups in comparison to the chow-fed groups (Figure 5a and Figure 6). However, *Desulfovibrio* expansion in HFD was enhanced ~1.2-fold in the HFX group. A study associated *Butyricimonas*, *Desulfovibrio*, and *Oscillospira* genera with a BMI < 25, that is, normal-weight people [56]. The possible discordance in the results can be attributed to the variability in the exercise methodology, selection of model organism, and choice of sequencing platform.

The random forest regression model has been reported to be one of the most effective supervised machine-learning algorithms for analyzing microbiome data [58]. The relative abundance of *Adlercreutzia* (*Coriobacteriaceae*, Actinobacteria) identified using random forest classification corroborates with the moderate expansion of *Coriobacteriaceae* at the family level in the high-fat diet-fed groups (Figure 4b). Moreover, exercise might have a role to play in the mild enhancement of *Oscillospira* (*Ruminococcaceae*) (Figure 5a, Figure 6), which might contribute to the mild amplification of its family *Ruminococcaceae* (Firmicutes), as implied by the results of the HFX group (Figure 4b). Consistent with our results, *Oscillospira* was found to be positively associated with the blood lactate levels in the exercised rats [59].

Faith’s phylogenetic diversity metric of alpha diversity and the unweighted UniFrac (qualitative, phylogenetic) metric of beta diversity were found to be insignificant among the groups, indicating that the experimental interventions did not significantly affect the phylogenetic diversity of the gut microbiome (data not shown). The HFD and HFX groups showed higher diversity (Shannon) and evenness than the CD and EX groups (Figure 2). This finding is supported by a recent study, which reported an increased gut microbial diversity as well as energy metabolism in mice fed an HFD for 14 weeks, compared to mice fed a low-fat diet for the same period. This was explained by studying the changes in the metabolic pathways. Specifically, the expression of genes involved in non-absorbed carbohydrate metabolism pathways were found to be increased in HFD mice, leading to the colonization of intestinal pathogens and inflammation [60].

Diet appeared to be a significant contributor to the alpha and beta diversities of the mouse gut microbiota, as examined by longitudinal ANOVA and ADONIS, when compared to exercise. Overall, the results showed a higher relative abundance of *Lactococcus* spp. in the guts of HFD mice than in those of CD mice (*p* < 0.0001). Interestingly, *Lactococcus* spp. abundance was significantly decreased in the HFX group compared to the HFD group (*p* = 0.0001) (Figure 5). The results of the present study are supported by a recent report showing the role of lactic acid bacteria in metabolic syndrome. In a previous study, yeast β-glucan was administered to HFD-induced metabolic syndrome mice for 10 weeks, which resulted in the reduction of the relative abundances of *Lactobacillus* and *Lactococcus*, explaining the possible attenuation of the metabolic syndrome in mice [61]. Since *Lactobacillus* and *Lactococcus* play a critical role in HFD-induced metabolic syndrome, the exercise-mediated decrease in the abundance of *Lactococcus* spp. shown by our data was encouraging. Further studies investigating the functional readout of the observed changes in the gut microbiota are required to better understand the physiological phenomena associated with different dietary or exercise interventions in the context of pathology and metabolic syndromes.

## 5. Conclusions

The present study revealed that variable diets in the context of fat percentage induces significant alterations in the gut microbiota composition of mice. In general, the gut microbiota were altered under different dietary interventions, while the microbial community richness was reduced as expected, but the Shannon diversity index increased in response to HFD. The B/F ratio significantly decreased in the high-fat diet-fed groups compared to the chow-fed controls. The relative abundance of bacteria belonging to Proteobacteria and Firmicutes was higher in the HFD group. *Lactococcus*, which was rare in the CD group and relatively abundant in the HFD group, was significantly reversed in abundance in the HFX group. A similar pattern was shown by the bacterial taxonomic family *Streptococcaceae*. Regardless of the comparably less significant effect of exercise, this study enabled us to resolve the taxonomic players at the genus level that contribute significantly to the changes observed at the phylum level in response to different diets. *Paraprevotella*, *Desulfovibrio*, *Lactococcus, Butyricimonas*, and *Desulfovibrio C21c20* were the differentially abundant/present taxa based on diet. From the results of this study, diet has emerged as a major contributor to the gut microbial richness and diversity in mice. These differential responses require detailed mechanistic studies to explore the relationship between the gut microbes and the dietary interventions (with or without exercise), by exploring the underlying players involved in bacterial metabolism and signaling.

## Figures and Tables

**Table 1:** Body weight and food intake of mice.

Group	Body Weight (g)			Food Intake (g/Day)
	Initial	Final	*p* Value (Final Weight) ^1^	
CD	10.5 ± 0.4	18.4 ± 1.1	a: <0.0001	15.6 ± 2.1
HFD	10.3 ± 0.7	30.5 ± 4	b: <0.001	9 ± 4.2
HFX	9.7 ± 0.8	25.6 ± 3.6	c: <0.005	8.5 ± 3.1
EX	10.2 ± 0.8	20.9 ± 0.8	-	12.1 ± 0.9

^1^ One-way ANOVA and Tukey’s multiple comparison test, a: CD/HFD, b: HFD/EX, c: CD/HFX, *n* = 5.

## Data Availability

The data generated during this study are available from NCBI under the accession number PRJNA846968.

## References

[1] Caballero B. (2005). A Nutrition Paradox — Underweight and Obesity in Developing Countries. New Engl. J. Med..

[2] World Health Organization (WHO), Geneva. Obesity and Overweight. http://www.who.int/news-room/fact-sheets/detail/obesity-and-overweight,2021.

[3] Rössner S. (2002). Obesity: The disease of the twenty-first century. Int. J. Obes..

[4] Schrauwen P., Westerterp K.R. (2000). The role of high-fat diets and physical activity in the regulation of body weight. Br. J. Nutr..

[5] Krauss R.M., Winston M., Fletcher B.J., Grundy S.M. (1998). Obesity: Impact on cardiovascular disease. Circulation.

[6] De Mello A.H., Costa A.B., Engel J.D.G., Rezin G.T. (2018). Mitochondrial dysfunction in obesity. Life Sci..

[7] Yang G., De Staercke C., Hooper W.C. (2012). The effects of obesity on venous thromboembolism: A review. Open J. Prev. Med..

[8] Chiller K., Selkin B.A., Murakawa G.J. (2001). Skin microflora and bacterial infections of the skin. Journal of Investigative Dermatology Symposium Proceedings.

[9] Neish A.S. (2009). Microbes in gastrointestinal health and disease. Gastroenterology.

[10] Aziz Q., Doré J., Emmanuel A., Guarner F., Quigley E. (2013). Gut microbiota and gastrointestinal health: Current concepts and future directions. Neurogastroenterol. Motil..

[11] Hooper L.V., Gordon J.I. (2001). Commensal host-bacterial relationships in the gut. Science.

[12] Sharon G., Garg N., Debelius J., Knight R., Dorrestein P.C., Mazmanian S.K. (2014). Specialized metabolites from the microbiome in health and disease. Cell Metab..

[13] Tilg H., Moschen A.R., Kaser A. (2009). Obesity and the microbiota. Gastroenterology.

[14] Lynch S.V., Pedersen O. (2016). The Human Intestinal Microbiome in Health and Disease. N. Engl. J. Med..

[15] Moreira A.B., Teixeira T.F.S., Alfenas R.D.C.G. (2012). Gut microbiota and the development of obesity. Nutr. Hosp..

[16] Falony G., Joossens M., Vieira-Silva S., Wang J., Darzi Y., Faust K., Kurilshikov A., Bonder M.J., Valles-Colomer M., Vandeputte D. (2016). Population-level analysis of gut microbiome variation. Science.

[17] McDonald D., Hyde E., Debelius J.W., Morton J.T., Gonzalez A., Ackermann G., Aksenov A.A., Behsaz B., Brennan C., Chen Y. (2018). American Gut: An Open Platform for Citizen Science Microbiome Research. Msystems.

[18] Rothschild D., Weissbrod O., Barkan E., Kurilshikov A., Korem T., Zeevi D., Costea P.I., Godneva A., Kalka I.N., Bar N. (2018). Environment dominates over host genetics in shaping human gut microbiota. Nature.

[19] Zhernakova A., Kurilshikov A., Bonder M.J., Tigchelaar E.F., Schirmer M., Vatanen T., Mujagic Z., Vila A.V., Falony G., Vieira-Silva S. (2016). Population-based metagenomics analysis reveals markers for gut microbiome composition and diversity. Science.

[20] Gilbert J.A., Blaser M.J., Caporaso J.G., Jansson J.K., Lynch S.V., Knight R. (2018). Current understanding of the human microbiome. Nat. Med..

[21] Kim H., Sitarik A.R., Woodcroft K., Johnson C.C., Zoratti E. (2019). Birth Mode, Breastfeeding, Pet Exposure, and Antibiotic Use: Associations With the Gut Microbiome and Sensitization in Children. Curr. Allergy Asthma Rep..

[22] Zhang C., Zhang M., Wang S., Han R., Cao Y., Hua W., Mao Y., Zhang X., Pang X., Wei C. (2010). Interactions between gut microbiota, host genetics and diet relevant to development of metabolic syndromes in mice. ISME J..

[23] Clauss M., Gérard P., Mosca A., Leclerc M. (2021). Interplay Between Exercise and Gut Microbiome in the Context of Human Health and Performance. Front. Nutr..

[24] Imdad S., Lim W., Kim J.-H., Kang C. (2022). Intertwined Relationship of Mitochondrial Metabolism, Gut Microbiome and Exercise Potential. Int. J. Mol. Sci..

[25] Liu W.-X., Wang T., Zhou F., Wang Y., Xing J.-W., Zhang S., Gu S.-Z., Sang L.-X., Dai C., Wang H.-L. (2015). Voluntary exercise prevents colonic inflammation in high-fat diet-induced obese mice by up-regulating PPAR-γ activity. Biochem. Biophys. Res. Commun..

[26] Clarke S.F., Murphy E.F., O′Sullivan O., Lucey A.J., Humphreys M., Hogan A., Hayes P., O′Reilly M., Jeffery I.B., Wood-Martin R. (2014). Exercise and associated dietary extremes impact on gut microbial diversity. Gut.

[27] Mailing L.J., Allen J.M., Buford T.W., Fields C.J., Woods J.A. (2019). Exercise and the Gut Microbiome: A Review of the Evidence, Potential Mechanisms, and Implications for Human Health. Exerc. Sport Sci. Rev..

[28] Durk R.P., Castillo E., Márquez-Magaña L., Grosicki G.J., Bolter N.D., Lee C.M., Bagley J.R. (2019). Gut microbiota composition is related to cardiorespiratory fitness in healthy young adults. Int. J. Sport Nutr. Exerc. Metab..

[29] Campbell S.C., Wisniewski P.J., Noji M., McGuinness L.R., Häggblom M.M., Lightfoot S.A., Joseph L.B., Kerkhof L.J. (2016). The Effect of Diet and Exercise on Intestinal Integrity and Microbial Diversity in Mice. PLoS ONE.

[30] Estaki M., Pither J., Baumeister P., Little J.P., Gill S.K., Ghosh S., Ahmadi-Vand Z., Marsden K.R., Gibson D.L. (2016). Cardiorespiratory fitness as a predictor of intestinal microbial diversity and distinct metagenomic functions. Microbiome.

[31] Evans C.C., LePard K.J., Kwak J.W., Stancukas M.C., Laskowski S., Dougherty J., Moulton L., Glawe A., Wang Y., Leone V. (2014). Exercise prevents weight gain and alters the gut microbiota in a mouse model of high fat diet-induced obesity. PLoS ONE.

[32] Kang S.S., Jeraldo P.R., Kurti A., Miller M.E., Cook M.D., Whitlock K., Goldenfeld N., Woods J.A., White B.A., Chia N. (2014). Diet and exercise orthogonally alter the gut microbiome and reveal independent associations with anxiety and cognition. Mol. Neurodegener..

[33] Lambert J.E., Myslicki J.P., Bomhof M.R., Belke D.D., Shearer J., Reimer R.A. (2015). Exercise training modifies gut microbiota in normal and diabetic mice. Appl. Physiol. Nutr. Metab..

[34] Dalton A., Mermier C., Zuhl M. (2019). Exercise influence on the microbiome-gut-brain axis. Gut Microbes.

[35] Bressa C., Bailén-Andrino M., Pérez-Santiago J., González-Soltero R., Pérez M., Montalvo-Lominchar M.G., Maté-Muñoz J.L., Domínguez R., Moreno D., Larrosa M. (2017). Differences in gut microbiota profile between women with active lifestyle and sedentary women. PLoS ONE.

[36] Hsu Y.J., Chiu C.C., Li Y.P., Huang W.C., Huang Y.T., Huang C.C., Chuang H.L. (2015). Effect of intestinal microbiota on exercise performance in mice. J. Strength Cond. Res..

[37] Bolyen E., Rideout J.R., Dillon M.R., Bokulich N.A., Abnet C.C., Al-Ghalith G.A., Alexander H., Alm E.J., Arumugam M., Asnicar F. (2019). Reproducible, interactive, scalable and extensible microbiome data science using QIIME 2. Nat. Biotechnol..

[38] Martin M. (2011). Cutadapt removes adapter sequences from high-throughput sequencing reads. EMBnet. J..

[39] Rognes T., Flouri T., Nichols B., Quince C., Mahé F. (2016). VSEARCH: A versatile open source tool for metagenomics. PeerJ.

[40] Pedregosa F., Varoquaux G., Gramfort A., Michel V., Thirion B., Grisel O., Blondel M., Prettenhofer P., Weiss R., Dubourg V. (2011). Scikit-learn: Machine Learning in Python. J. Mach. Learn. Res..

[41] McDonald D., Price M.N., Goodrich J., Nawrocki E.P., DeSantis T.Z., Probst A., Andersen G.L., Knight R., Hugenholtz P. (2012). An improved Greengenes taxonomy with explicit ranks for ecological and evolutionary analyses of bacteria and archaea. ISME J..

[42] Bokulich N.A., Dillon M.R., Bolyen E., Kaehler B.D., Huttley G.A., Caporaso J.G. (2018). q2-sample-classifier: Machine-learning tools for microbiome classification and regression. J. Open Res. Softw..

[43] Buettner R., Schölmerich J., Bollheimer L.C. (2007). High-fat diets: Modeling the metabolic disorders of human obesity in rodents. Obesity.

[44] Magne F., Gotteland M., Gauthier L., Zazueta A., Pesoa S., Navarrete P., Balamurugan R. (2020). The Firmicutes/Bacteroidetes Ratio: A Relevant Marker of Gut Dysbiosis in Obese Patients?. Nutrients.

[45] Stojanov S., Berlec A., Štrukelj B. (2020). The Influence of Probiotics on the Firmicutes/Bacteroidetes Ratio in the Treatment of Obesity and Inflammatory Bowel disease. Microorganisms.

[46] Krajmalnik-Brown R., Ilhan Z.E., Kang D.W., DiBaise J.K. (2012). Effects of gut microbes on nutrient absorption and energy regulation. Nutr. Clin. Pr..

[47] Ley R.E., Bäckhed F., Turnbaugh P., Lozupone C.A., Knight R.D., Gordon J.I. (2005). Obesity alters gut microbial ecology. Proc. Natl. Acad. Sci. USA.

[48] Turnbaugh P.J., Ley R.E., Mahowald M.A., Magrini V., Mardis E.R., Gordon J.I. (2006). An obesity-associated gut microbiome with increased capacity for energy harvest. Nature.

[49] Koliada A., Syzenko G., Moseiko V., Budovska L., Puchkov K., Perederiy V., Gavalko Y., Dorofeyev A., Romanenko M., Tkach S. (2017). Association between body mass index and Firmicutes/Bacteroidetes ratio in an adult Ukrainian population. BMC Microbiol..

[50] Ms E.Y.H., Leone V.A., Devkota S., Wang Y., Brady M.J., Chang E.B. (2013). Composition of Dietary Fat Source Shapes Gut Microbiota Architecture and Alters Host Inflammatory Mediators in Mouse Adipose Tissue. J. Parenter. Enter. Nutr..

[51] Shin N.R., Whon T.W., Bae J.W. (2015). Proteobacteria: Microbial signature of dysbiosis in gut microbiota. Trends Biotechnol..

[52] Mulkidjanian A.Y., Koonin E.V., Makarova K.S., Mekhedov S.L., Sorokin A., Wolf Y.I., Dufresne A., Partensky F., Burd H., Kaznadzey D. (2006). The cyanobacterial genome core and the origin of photosynthesis. Proc. Natl. Acad. Sci. USA.

[53] Di Rienzi S.C., Sharon I., Wrighton K.C., Koren O., Hug L.A., Thomas B.C., Goodrich J.K., Bell J.T., Spector T.D., Banfield J.F. (2013). The human gut and groundwater harbor non-photosynthetic bacteria belonging to a new candidate phylum sibling to Cyanobacteria. eLife.

[54] Panasevich M.R., Wankhade U.D., Chintapalli S.V., Shankar K., Rector R.S. (2018). Cecal versus fecal microbiota in Ossabaw swine and implications for obesity. Physiol. Genom..

[55] Li K., Liu A., Zong W., Dai L., Liu Y., Luo R., Ge S., Dong G. (2021). Moderate exercise ameliorates osteoarthritis by reducing lipopolysaccharides from gut microbiota in mice. Saudi J. Biol. Sci..

[56] Garcia-Mantrana I., Selma-Royo M., Alcantara C., Collado M.C. (2018). Shifts on Gut Microbiota Associated to Mediterranean Diet Adherence and Specific Dietary Intakes on General Adult Population. Front. Microbiol..

[57] Zeng H., Ishaq S.L., Zhao F.-Q., Wright A.-D.G. (2016). Colonic inflammation accompanies an increase of β-catenin signaling and Lachnospiraceae/Streptococcaceae bacteria in the hind gut of high-fat diet-fed mice. J. Nutr. Biochem..

[58] Statnikov A., Henaff M., Narendra V., Konganti K., Li Z., Yang L., Pei Z., Blaser M.J., Aliferis C.F., Alekseyenko A.V. (2013). A comprehensive evaluation of multicategory classification methods for microbiomic data. Microbiome.

[59] Petriz B.A., Castro A.P., Almeida J.A., Gomes C.P.C., Fernandes G.R., Kruger R.H., Pereira R.W., Franco O.L. (2014). Exercise induction of gut microbiota modifications in obese, non-obese and hypertensive rats. BMC Genom..

[60] Wang B., Kong Q., Li X., Zhao J., Zhang H., Chen W., Wang G. (2020). A High-Fat Diet Increases Gut Microbiota Biodiversity and Energy Expenditure Due to Nutrient Difference. Nutrients.

[61] Chen G., Chen D., Zhou W., Peng Y., Chen C., Shen W., Zeng X., Yuan Q. (2021). Improvement of Metabolic Syndrome in High-Fat Diet-Induced Mice by Yeast β-Glucan Is Linked to Inhibited Proliferation of Lactobacillus and Lactococcus in Gut Microbiota. J. Agric. Food Chem..

